# Cost-effectiveness of antibiotic treatment strategies for community-acquired pneumonia: results from a cluster randomized cross-over trial

**DOI:** 10.1186/s12879-016-2179-6

**Published:** 2017-01-10

**Authors:** Cornelis H. van Werkhoven, Douwe F. Postma, Marie-Josee J. Mangen, Jan Jelrik Oosterheert, Marc J. M. Bonten, for the CAP-START study group

**Affiliations:** 1 Julius Center for Health Sciences and Primary Care, University Medical Center Utrecht, Utrecht, The Netherlands; 2 Department of Internal Medicine and Infectious Diseases, University Medical Center Utrecht, Heidelberglaan 100, 3584 CX Utrecht, The Netherlands; 3 Department of Internal Medicine, Diakonessenhuis Utrecht, Utrecht, The Netherlands; 4 Department of Medical Microbiology, University Medical Center Utrecht, Utrecht, The Netherlands

**Keywords:** Beta-lactam macrolide, Fluoroquinolone, Cost-effectiveness, Community acquired pneumonia

## Abstract

**Background:**

To determine the cost-effectiveness of strategies of preferred antibiotic treatment with beta-lactam/macrolide combination or fluoroquinolone monotherapy compared to beta-lactam monotherapy.

**Methods:**

Costs and effects were estimated using data from a cluster-randomized cross-over trial of antibiotic treatment strategies, primarily from the reduced third payer perspective (i.e. hospital admission costs). Cost-minimization analysis (CMA) and cost-effectiveness analysis (CEA) were performed using linear mixed models. CMA results were expressed as difference in costs per patient. CEA results were expressed as incremental cost-effectiveness ratios (ICER) showing additional costs per prevented death.

**Results:**

A total of 2,283 patients were included. Crude average costs within 90 days from the reduced third payer perspective were €4,294, €4,392, and €4,002 per patient for the beta-lactam monotherapy, beta-lactam/macrolide combination, and fluoroquinolone monotherapy strategy, respectively. CMA results were €106 (95% CI €-697 to €754) for the beta-lactam/macrolide combination strategy and €-278 (95%CI €-991 to €396) for the fluoroquinolone monotherapy strategy, both compared to the beta-lactam monotherapy strategy. The ICER was not statistically significantly different between the strategies. Other perspectives yielded similar results.

**Conclusions:**

There were no significant differences in cost-effectiveness of strategies of preferred antibiotic treatment of CAP on non-ICU wards with either beta-lactam monotherapy, beta-lactam/macrolide combination therapy, or fluoroquinolone monotherapy.

**Trial registration:**

The trial was registered with ClinicalTrials.gov, number NCT01660204, on May 2nd, 2012.

**Electronic supplementary material:**

The online version of this article (doi:10.1186/s12879-016-2179-6) contains supplementary material, which is available to authorized users.

## Background

Community-acquired pneumonia (CAP) is an important reason for hospitalization worldwide [1–3]. It has been estimated that the total costs associated with CAP amount to approximately 11 billion euros annually in Europe, with approx. 5 billion euros accounting for in-hospital CAP costs [1]. In the Netherlands there are an estimated 25,000-36,000 hospital admissions for CAP each year, [4] with an estimated total costs of about 100 to 178 million euro annually [5, 6]. The intramural costs are mainly determined by the length of hospitalization and site of care (medical ward or intensive care unit, ICU) [5, 6].

In choosing the optimal antibiotic treatment strategy for CAP, effectiveness, cost-effectiveness and ecological effects of antibiotics should be taken into account. Optimally, this would consist of a strategy associated with the best patient outcome at the lowest price and with least selective pressure for antibiotic resistance. The three treatment strategies most widely used are beta-lactam monotherapy, beta-lactam/macrolide combination therapy, and fluoroquinolone monotherapy. From an ecological perspective beta-lactam monotherapy is preferred over beta-lactam/ macrolide combination therapy, and fluoroquinolone monotherapy, since the latter two drug classes have been associated with resistance development during treatment [7, 8].

In a cluster-randomized cross-over trial of patients hospitalized with CAP to non-ICU wards, a strategy of beta-lactam monotherapy was non-inferior to beta-lactam/macrolide combination therapy, and fluoroquinolone monotherapy in terms of all-cause day-90 mortality (CAP-START study) [9]. The quinolone monotherapy strategy was associated with a shorter length of intravenous treatment, but this was not reflected in a statistically significant shorter length of stay. In the current study, we set out to conduct a cost-minimization analysis of these different antibiotic strategies and a cost-effectiveness analysis from a third payer and a social perspective.

## Methods

### Intervention

The Community-Acquired Pneumonia Study on the initial Treatment with Antibiotics of Lower Respiratory Tract Infections (CAP-START, http://clinicaltrials.gov/show/NCT01660204) was a cluster-randomized cross-over trial that was performed in seven hospitals in the Netherlands between February 2011 and August 2013. Details of the study design, enrolment, and clinical outcomes have been published previously [9, 10]. In short, three strategies were compared in which one class or combination of antibiotics (beta-lactam monotherapy, beta-lactam/macrolide combination therapy or fluoroquinolone monotherapy) was the preferred empirical treatment for adult patients hospitalized to non-intensive care unit (ICU) wards with a clinical diagnosis of CAP. Hospitals were randomized to a sequence of consecutive periods of 4 months, in each of which one of the strategies were applied. Deviations from the preferred empirical treatment for medical reasons were allowed, e.g. because of contra-indications, allergy to the preferred regimen, or a suspected pathogen not covered by the preferred regimen. Physicians were encouraged to complete the preferred empirical treatment unless for a medical reason, e.g. insufficient recovery or deterioration of the patient, or detection of a pathogen for which targeted antibiotic treatment was initiated. Based on an intention-to-treat principle, inclusion of patients was independent of compliance with the strategy, which allowed us to assess the effect of the strategy as a whole.

### Effects

For health outcomes we used 30- and 90-day all-cause mortality, which have been reported previously [9]. Mortality status at day 90 was recorded from the medical charts in patients that died during hospitalization, and patients that had visited the hospital after day 90 (e.g. in an out-patient clinic). The status of all other patients, except in one hospital, was checked electronically in the municipal personal records database, which is based on the citizen service number, date of birth and name. In the one hospital without electronic access to this database, research nurses contacted the general practitioner of each patient with an unknown status. In the Netherlands, every inhabitant is registered with a single general practitioner, who is routinely informed about important medical affairs.

### Cost of illness

Data on healthcare resource utilization during hospitalization, e.g. hospital days, interventions, and medication (see Additional file 1: Table S2 for a complete overview), were derived from the medical records by trained research nurses using a predefined clinical record form. For other resources, patients were asked to complete a questionnaire on the 28^th^ day after admission. This 28^th^ day questionnaire included questions on post-discharge healthcare use such as nursing home admission, general practitioner and specialist consultations, patient costs (e.g. travel costs), and the number of days absent from paid and unpaid work for both patients and their caregivers. We defined caregivers as adult persons taking absence from paid or unpaid work in order to take care of a sick person.

Direct healthcare costs (DHC), direct non-healthcare costs (DNHC) - also referred to as patient costs -, and productivity losses (i.e. indirect non-healthcare costs-INHC) were considered in the current study. In accordance with the current Dutch guidelines for health economic evaluations, this study did not consider indirect healthcare costs [11, 12]. Indirect healthcare costs would comprise the future savings in healthcare costs in the life years lost due to premature death. DHC were composed of healthcare costs related to hospitalization, e.g. days admitted to non-ICU wards, ICU days with and without mechanical ventilation, medical interventions, antibiotic use, other medication use, and post-discharge healthcare consumption. In the DNHC category, travel costs to a general practitioner (GP), to a hospital, or over-the-counter medication were considered. Productivity losses were estimated for non-fatal CAP cases by multiplying self-reported sick leave from paid and unpaid work with the corresponding age and gender specific unit prices as reported in Additional file 1: Table S1. For fatal cases younger than 65 years, two approaches were used: the friction and the human capital approach. The friction approach, recommended in Dutch guidelines, takes into account the productivity loss from paid work due to case fatality for a period of 23 weeks from the date of admission [11, 12]. In the human capital approach, productivity losses from work due to case fatality up to the age of retirement were considered, leading to higher costs due to productivity loss for deceased patients under 65 years of age.

Costs were estimated by multiplying resources used with their corresponding unit cost prices. Additional file 1: Table S1 depicts unit cost prices for all DHC, DNHC, and INHC used in the analyses. All costs are expressed in 2012 euros and, if necessary, updated using Dutch consumer price indexes [4].

Two time horizons of 30 and 90 days were used for the economic evaluation, in accordance with the time horizons used for the effects under study, i.e. 30-day and 90-day mortality [9]. Hospital and nursing home admission costs were calculated until discharge or until the time horizon, whichever came first. For productivity losses from case-fatality, deaths falling within the defined time horizon were used, but, as explained previously, costs were extended to 23 weeks using the friction approach [11, 12], and to retirement age using the human capital approach, respectively. Discounting was only applied for productivity losses longer than 1 year (i.e. the human capital approach), using a 3% annual discount rate [13]. As in the primary analysis of clinical outcomes, the 90-day time horizon was considered for the primary analysis.

### Economic evaluation

Cost-minimization analysis (CMA) and cost-effectiveness analysis (CEA) were conducted using four different perspectives. The “reduced” third payer perspective included only DHC of the CAP hospitalization. This perspective constituted the primary analysis of medical records, and as such healthcare utilization data during admission, were available for all patients. The “full” third payer perspective (referred hereafter as third payer perspective) included both DHC during admission and post-discharge. The societal perspective considered all three categories (i.e. DHC, DNHC and INHC). Two approaches were used here, the friction and the human capital approach, as explained previously.

The beta-lactam monotherapy strategy was considered the reference arm, as this is considered the first choice treatment for patients hospitalized with CAP to non-ICU wards in the Netherlands [14]. As the primary outcome of the CAP-START trial, i.e. prevented deaths per treated person, was not statistically significantly different between the strategies [9], we conducted a CMA, assessing the incremental costs per treated case. Additionally, because small effects on clinical outcomes could not be excluded, a CEA was conducted showing the incremental costs (or savings) of the net effect (i.e. number of deaths prevented), expressed as incremental cost-effectiveness ratio (ICER) showing additional costs per prevented death.

### Data analysis

Crude average costs were calculated for each antibiotic treatment strategy. For calculating incremental costs, we adjusted for the cluster-randomized design of the study, by using a mixed-effects linear regression analysis, with a random intercept for each cluster-period of 4 months, and fixed effects for hospital and treatment arm. A random intercept is used in mixed-effect models to allow for dependence of observations within one cluster [15]. For cost-minimization and cost-effectiveness analyses, differences in mortality (i.e. the incremental effect) were assessed similarly using a mixed-effects logistic regression analysis. We performed bootstrapping with 2,000 samples to obtain confidence intervals. For missing values, five imputations were performed in each bootstrapped dataset. In each of the imputed datasets, the costs and effects were compared between the treatment strategies using the aforementioned mixed-effects models. Incremental costs and effects were averaged over these 5 imputations, again resulting in 2,000 estimates of incremental costs and effects. From these, we derived incremental costs and effects which were presented as cost-effectiveness plots. 95% confidence intervals were derived from these estimates using the quantile method. Significance for cost-minimization and cost-effectiveness was defined as a 95% confidence interval not covering the null effect.

## Results

### Patient, data collection, and missing data

In total 656, 739, and 888 patients were included during the beta-lactam, beta-lactam/macrolide and fluoroquinolone strategies. Age, gender, and comorbidities had similar distributions in the three treatment arms (Table 1). Inclusion rates, strategy adherence, and reasons for protocol deviations and switches have been described previously [9]. Response rates for the self-reported 28^th^ day questionnaire were comparable in all three treatment arms (42.1%, 34.2%, and 42.3% for beta-lactam monotherapy, beta-lactam/macrolide combination, and fluoroquinolone monotherapy strategy respectively).

**Table 1:** Baseline characteristics

	Beta-lactam monotherapy (*N* = 656)	Beta-lactam/macrolide (*N* = 739)	Fluoroquinolone monotherapy (*N* = 888)
Median age (IQR)	70.6 (60.6–79.4)	70.7 (59.1–80.3)	71.0 (59.6–79.4)
Male gender	381 (58.1%)	431 (58.3%)	505 (56.9%)
Elderly home	32 / 644 (5.0%)	38 / 727 (5.2%)	41 / 878 (4.7%)
Hospitalization past 12 months	271 / 653 (41.5%)	298 / 722 (41.3%)	351 / 881 (39.8%)
Median number of comorbidities (IQR) ^a^	1 (0–2)	1 (0–2)	1 (1–2)
Immunocompromised ^b^	147 (22.4%)	173 (23.4%)	213 (24.0%)
Median CURB-65 score (IQR) ^d^	1 (1–2)	1 (1–2)	1 (1–2)
Day-28 questionnaire received	276 (42.1%)	253 (34.2%)	376 (42.3%)
Reports paid work	51 / 246 (20.7%)	45 / 233 (19.3%)	78 / 342 (22.8%)
Reports volunteer work	23 / 245 (9.4%)	32 / 234 (13.7%)	35 / 340 (10.3%)

Data are reported as N (%) unless otherwise indicated. IQR: inter quartile range

^a^ Reported comorbidities include chronic cardiovascular disease, heart failure, cerebrovascular disease, asthma, COPD, other chronic pulmonary disease, HIV/AIDS, diabetes mellitus, haematological malignancies^c^, solid organ malignancies^c^, chronic renal failure requiring dialysis, nephrotic syndrome, organ or bone marrow transplantation, alcoholism, chronic liver disease and functional or anatomic asplenia

^b^Patients were categorized as immunocompromised if any of the following conditions applied: HIV/AIDS, haematological malignancies#, solid organ malignancies^c^, chronic renal failure requiring dialysis, nephrotic syndrome, organ or bone marrow transplantation, or receipt of immunosuppressive therapy (for corticosteroids this required at least 0.5 mg/kg/day prednisolone or equivalent dosage for a minimum of 14 days)

^c^ Having received or been eligible for chemotherapy or radiotherapy in the past 5 years

^d^ The CURB-65 score is calculated by assigning 1 point each for confusion, uraemia (blood urea nitrogen ≥20 mg per deci- liter), high respiratory rate (≥30 breaths per minute), low systolic blood pressure (<90 mm Hg) or diastolic blood pres- sure (≤60 mm Hg), and an age of 65 years or older, with a higher score indicating a higher risk of death within 30 days

In total, 2.1 and 6.6% of data points from the medical records and received 28^th^ day questionnaires, respectively, were missing.

### Cost of illness and economic evaluation

**Fig. 1:**
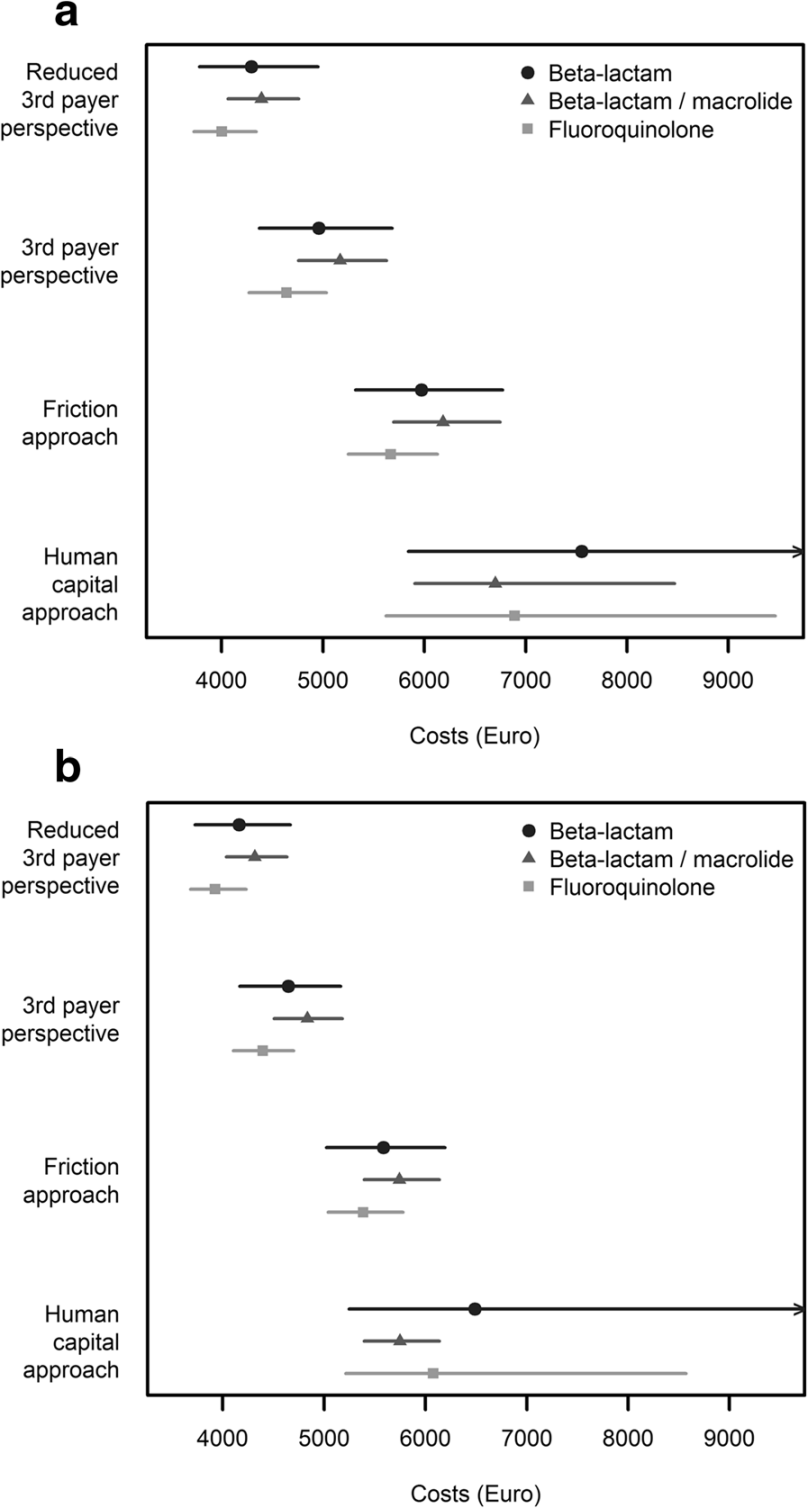
Mean costs per patient. **a** 90-day time horizon. **b** 30-day time horizon. Legend: Mean costs per patient for the three treatment strategies taking four different perspectives and applying a 90-day (**a**) and 30-day (**b**) time horizon. Point estimates and confidence intervals are generated using the 50^th^, 2.5^th^ and 97.5^th^ percentiles of 2,000 bootstrapping samples. Exact numbers are given in Additional file 1: Table S3

Crude (i.e. not adjusted for the cluster-randomized cross-over design) average costs within 90 days from the reduced third payer perspective (i.e. hospitalization costs) were €4,294 (95% confidence interval, CI €3,782 to €4,952) per patient for the beta-lactam monotherapy strategy, €4,392 (95% CI €4,062 to €4,760) per patient for the beta-lactam/macrolide combination strategy, and €4,002 (95% CI €3,725 to €4,341) per patient for the fluoroquinolone monotherapy strategy (Fig. 1). For the CMA using the reduced third payer perspective within the 90-day time horizon, estimated incremental costs, adjusted for cluster and period effects using a mixed-effects model, were €106 (95% CI -€697 to €754) per patient for the beta-lactam/macrolide combination strategy and -€278 (95%CI -€991 to €396) for the fluoroquinolone monotherapy strategy, a positive number indicating higher costs as compared to the beta-lactam monotherapy strategy.

**Fig. 2:**
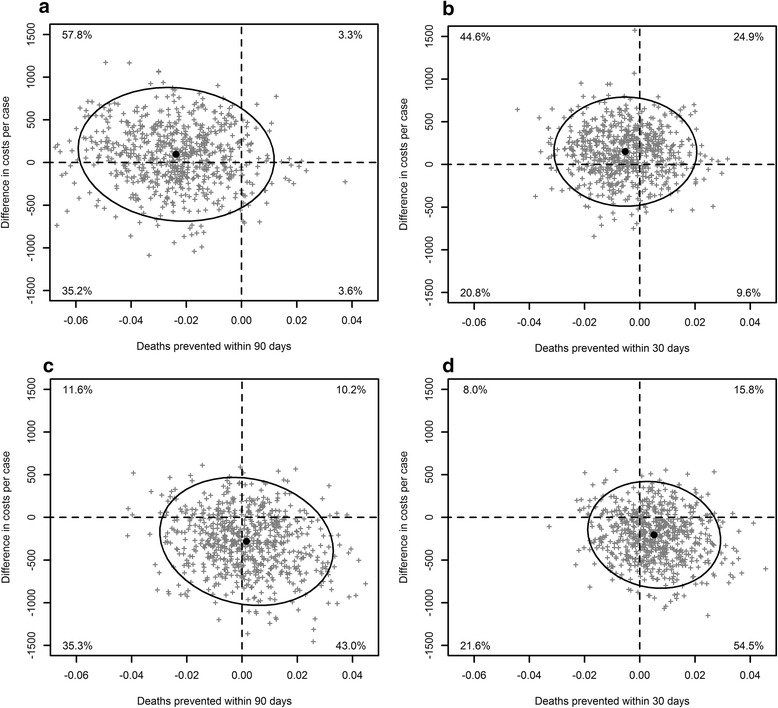
Cost-effectiveness plots from a reduced third payer perspective. **a** Beta-lactam/macrolide strategy vs. beta-lactam strategy-90-day time horizon. **b** Beta-lactam/macrolide strategy vs. beta-lactam strategy-30-day time horizon. **c** Fluoroquinolone monotherapy strategy vs. beta-lactam strategy-90-day time horizon. **d** Fluoroquinolone monotherapy strategy vs. beta-lactam strategy-30-day time horizon. Legend: Grey points represent incremental costs and incremental effects of 2,000 bootstrapping samples for the beta-lactam/macrolide combination strategy compared to the beta-lactam monotherapy strategy within 90 (**a**) and 30 (**b**) days of admission, and for the fluoroquinolone monotherapy strategy compared to the beta-lactam monotherapy strategy within 90 (**c**) and 30 (d) days of admission. The black points and curves represent the point estimates and the 95% confidence ellipses. Proportions in each quadrant indicate the proportion of bootstrap samples in that quadrant. Point estimates in the north-west quadrant are in favour of the beta-lactam monotherapy strategy; point estimates in the south-east quadrant are in favour of the other strategy. Exact point estimates and 95% confidence intervals for incremental costs and incremental effects are given in Additional file 1: Table S3

For the beta-lactam/macrolide strategy compared to the beta-lactam strategy, using the reduced third payer perspective and the 90-day time horizon, 57.8% of the bootstrap results was in the north-west quadrant (i.e. positive incremental costs, the beta-lactam/macrolide strategy was more costly than the beta-lactam strategy, and negative incremental effects, the beta-lactam/macrolide strategy prevented fewer deaths than the beta-lactam strategy, thus beta-lactam dominates the beta-lactam/macrolide strategy), 3.3% was in the north-east quadrant (i.e. “positive” incremental costs and a positive incremental effect), 35.2% was in the south-west quadrant (i.e. negative incremental costs or cost-savings and a negative incremental effect), and 3.6% was in the south-east quadrant (i.e. negative incremental costs and a positive incremental effect), with the point estimate for the ICER in the north-west quadrant (Fig. 2a). For the fluoroquinolone strategy compared to the beta-lactam strategy, using the same perspective and time window, 11.6% was in the north-west quadrant, 10.2% in the north-east quadrant, 35.3% in the south-west quadrant, and 43.0% was in the south-east quadrant, with the point estimate for ICER in the south-east quadrant (Fig. 2c). Thus, the 95% confidence interval of the ICER ranged from being dominated (positive incremental costs and negative incremental effect) to cost-saving (negative incremental costs or savings and positive incremental effects or more prevented deaths) for both comparisons.

Similar results for costs, CMA, and CEA were obtained for the third payer perspective and for the societal perspective taking the friction approach (Fig. 2, Additional file 1: Figure S1, Figure S2, and Table S3), as well as for the 30-day time horizon for these three perspectives. The societal perspective with human capital approach had large confidence intervals for costs, for both time horizons, leading to uninterpretable results for both CMA and CEA (Additional file 1: Figure S3 and Table S3).

## Discussion

In these analyses, we have demonstrated that the differences in costs associated with either of three preferred empirical antibiotic treatment strategies (i.e., beta-lactam monotherapy, beta-lactam/macrolide combination therapy, or fluoroquinolone monotherapy) for patients hospitalized for community-acquired pneumonia did not reach statistical significance. Together with non-inferiority of the beta-lactam monotherapy strategy for day-90 mortality [9] and the perceived preference of beta-lactam monotherapy from an ecological perspective, the current analysis supports the use of beta-lactam monotherapy as preferred empirical treatment for these patients.

This is the first comparison of costs and cost-effectiveness for different preferred antibiotic treatment strategies in patients hospitalized with CAP. Our study has several strengths. First, because this was a pragmatic trial, where patients were included during strategy periods regardless of the actual antibiotics used, the intention-to-treat analysis of our study is well generalizable to daily clinical practice. All patients that received antibiotic treatment for a working diagnosis of CAP and who were hospitalized to a non-ICU medical ward, were eligible. Second, the cluster-randomized design allowed the immediate start of the allocated antibiotic treatment because individual randomization was not needed. This minimizes effects of other antibiotics prescribed in the Emergency Departments before study randomization. Third, because of the cross-over design, all hospitals applied all three strategies, thus minimizing confounding bias. As a result, baseline characteristics of the three strategies were very comparable. Fourth, we have collected comprehensive data on antibiotic treatment and medical procedures that allowed us to estimate hospitalization costs per patient. Using 2,000 bootstrapping samples and five imputations per sample, we were able to provide robust estimates and confidence intervals for the different cost categories. Our estimated costs per CAP admission are in line with previously published data from the Netherlands [5, 6]. Fifth, different economic viewpoints were pursued in the current analysis. The (reduced) third payer perspective and the societal perspective taking the friction approach all gave the same direction and magnitude of effect. The large confidence intervals observed in the societal perspective with human capital approach was due to the low number of fatal cases under 65 years of age and due to working status being unknown for unreturned 28^th^ day questionnaires. This led to unstable imputation of working status, since these variables also interact i.e. the proportion of returned questionnaires was lower for patients that had died at day 90, thus increasing confidence intervals.

Our approach had certain limitations. We had limited data on medication use other than antibiotics. Although it seems unlikely that one of the antibiotic treatment strategies would be associated with other patterns of non-antibiotic medication use, if so, we may have slightly underestimated the costs. 28^th^ day questionnaires, used for DNHC and INHC estimation, were returned by approximately 40% of the participants. We used multiple imputation to deal with missing data because response to the 28^th^ day questionnaire was obviously dependent on clinical outcome and was related to baseline characteristics (e.g. dependency in activities of daily living or hospitalizations in the previous year). This may have increased uncertainty for the third payer and societal perspectives, and it certainly did for the societal perspective with human capital approach, as explained previously.

The number of days on intravenous antibiotic treatment was significantly lower during the fluoroquinolone monotherapy strategy (hazard ratio for time to switch to oral treatment 1.29, 95% CI 1.15–1.46) [9]. This was fully explained by the larger proportion of patients starting with oral treatment from the day of admission, despite the similar baseline characteristics between the different strategies, and can, therefore, not be attributed to a faster clinical response. The known high bioavailability of oral fluoroquinolones [16] may have stimulated physicians to directly start with oral antibiotics and this may have contributed to the more favourable point estimate of difference in costs seen in the fluoroquinolone monotherapy period. Whether the same proportion of patients could start with oral beta-lactam monotherapy without compromising patient outcome remains to be elucidated.

In an open-label randomized controlled trial from Switzerland, beta-lactam monotherapy was not non-inferior to beta-lactam/macrolide combination therapy in establishing clinical stability after seven days of antibiotic treatment [17]. This study was not designed to determine non-inferiority for day-30 or day-90 mortality, and there were no statistically significant or clinically relevant differences in outcome between both study arms. Time to clinical stability was not determined in our study, however, length of stay was significantly longer for the beta-lactam/macrolide combination strategy, and consequently also the costs per patient were higher, although not statistically significant. This seemingly opposite finding might in part be explained by the maximized adherence to the allocated antibiotic, i.e. the strict criteria for switching antibiotic treatment, which could only have disadvantaged the beta-lactam monotherapy arm in the Swiss study. The current analysis shows that any benefit of beta-lactam/macrolide combination treatment on time to clinical stability, if present, does not lead to cost reduction.

Generalizability of the estimated costs may depend on several factors, the most important of which are the duration of hospitalization, ICU admission, the length of intravenous and oral antibiotics, and post discharge patterns of healthcare use. Although the actual reported costs are obviously specific for the Netherlands, the relative differences in costs for medication are comparable internationally [18, 19]. As the generalizability of clinical outcome may depend on the proportion of CAP caused by pathogens not covered by beta-lactam monotherapy, as discussed previously [9], we think that the cost-efficacy will be similar in most regions with comparable etiology.

## Conclusions

In conclusion, there is no significant difference in cost-effectiveness of a strategy of preferred beta-lactam monotherapy compared to beta-lactam/macrolide combination therapy or fluoroquinolone monotherapy for the empirical antibiotic treatment of CAP in non-ICU wards. Together with the preference of narrow-spectrum antibiotics from an ecological perspective, these data support the use of beta-lactam monotherapy as preferred empirical treatment for patients hospitalized with community-acquired pneumonia.

## Additional file

Additional file 1: Table S1. Cost unit prices. **Table S2.** Resources used. **Table S3.** Cost and effect estimates and cost-effectiveness ratios. **Figure S1.** Cost-effectiveness plots-Third payer perspective. **Figure S2.** Cost-effectiveness plots-Societal perspective, friction approach. **Figure S3.** Cost-effectiveness plots-Societal perspective, human capital approach (DOCX 1332 kb)

## Abbreviations

CAP: Community-acquired pneumonia
CAP-START: Community-Acquired Pneumonia Study on the initial Treatment with Antibiotics of Lower Respiratory Tract Infections, http://clinicaltrials.gov/show/NCT01660204
CEA: Cost-effectiveness analysis
CER: Cost-effectiveness ratios
CMA: Cost-minimization analysis
DHC: Direct healthcare costs
DNHC: Direct non-healthcare costs
GP: General practitioner
ICU: Intensive Care Unit
INHC: Indirect non-healthcare costs
