# Supplementary material for: Cost-effectiveness of antibiotic treatment strategies for community-acquired pneumonia: results from a cluster randomized cross-over trial
Source: BMC Infect Dis. 2017 Jan 10;17:52. doi: 10.1186/s12879-016-2179-6 (PMC5223446; doi:10.1186/s12879-016-2179-6)
Supplement: Additional file 1: Table S1. — Cost unit prices. Table S2. Resources used. Table S3. Cost and effect estimates and cost-effectiveness ratios. Figure S1. Cost-effectiveness plots-Third payer perspective. Figure S2. Cost-effectiveness plots-Societal perspective, friction approach. Figure S3. Cost-effectiveness plots-Societal perspective, human capital approach (DOCX 1332 kb) [file 12879_2016_2179_MOESM1_ESM.docx]

**Supplementary Appendix**

**Table of contents**

List of investigators for the CAP-START study group

Table S1: Cost unit prices

Table S2: Resources used

Table S3: Cost and effect estimates and cost-effectiveness ratios

Figure S1: Cost-effectiveness plots – Third payer perspective

Figure S2: Cost-effectiveness plots – Societal perspective, friction approach

Figure S3: Cost-effectiveness plots – Societal perspective, human capital approach

**List of investigators for the CAP-START study group**

*University Medical Center Utrecht*

C.H. van Werkhoven, MD Julius Center for Health Sciences and Primary Care

D.F. Postma, MD Julius Center for Health Sciences and Primary Care

Department of Internal Medicine and Infectious Diseases

F. Teding van Berkhout, MD PhD Department of pulmonology

A.I.M. Hoepelman, MD PhD Department of Internal Medicine and Infectious Diseases

J.J. Oosterheert, MD PhD Department of Internal Medicine and Infectious Diseases

M.J.M. Bonten, MD PhD Department of Medical Microbiology

Julius Center for Health Sciences and Primary Care

*Diakonessenhuis Utrecht*

D.F. Postma, MD Department of Internal Medicine

S.F.T. Thijsen, MD PhD Department of Medical Microbiology

S.U.C. Sankatsing, MD PhD Department of internal medicine

L.J.R. van Elden, MD PhD Department of Pulmonology

*Amphia Ziekenhuis Breda*

J.A.J.W. Kluytmans, MD PhD Department of medical microbiology

J.G.J.V. Aerts, MD PhD Department of pulmonology

*Medisch Centrum Alkmaar*

W.G. Boersma, MD, PhD Department of pulmonology

*Kennemer Gasthuis Haarlem*

C.J. Compaijen, MD Department of internal medicine

R. Soetekouw, MD Department of internal medicine

*Spaarne Ziekenhuis Hoofddorp*

R.H. veenhoven, MD PhD † Research Centre Linnaeus Institute

E. van der Wall, MD Department of pulmonology

I. van der Lee, MD PhD Department of pulmonology

*Academic Medical Center Amsterdam*

J.M. Prins, MD PhD Department of internal medicine

R.E. Jonkers, MD PhD Department of pulmonology

**Table S1: Unit cost prices in 2012 € and underlying assumptions**

| **Variable** | **Unit cost** | **Source** |
| --- | --- | --- |
| ***Direct healthcare costs (DHC)*** |  |  |
| ***Pharmacy costs*** |  |  |
| Pharmacy fee for delivery per prescription | 5.80 | a |
| Additional pharmacy fee for first delivery | 3.23 | a |
| ***Overhead costs per antibiotic dosage*** |  |  |
| Nursery costs | 0.90 | b |
| Material costs | 7.64 | b |
| ***Antibiotics per antibiotic dosage*** |  |  |
| penicillin iv (6 dd. 1 x10^6^. IE) | 0.88 | c, ^1^ |
| amoxicillin iv (3 dd. 1000mg) | 1.37 | c, ^1^ |
| amoxicillin oral (3 dd. 500mg) | 0.09 | c, ^1^ |
| amoxicillin/clavulanic acid iv (3 dd. 1000/200mg) | 3.25 | c, ^1^ |
| amoxicillin/clavulanic acid oral (3 dd. 500/125mg) | 0.39 | c, ^1^ |
| cefuroxime iv (3 dd. 750mg) | 2.9 | c, ^1^ |
| cefuroxime axetil oral (3 dd. 250mg) | 0.59 | c, ^1^ |
| ceftriaxone iv (1 dd. 2g) | 17.625 | c, ^1^ |
| cefotaxime iv (2 dd. 1g) | 5.94 | c, ^1^ |
| ceftazidime iv (3 dd. 1g) | 8.91 | c, ^1^ |
| cefazoline iv (2 dd. 1g) | 2.88 | c, ^1^ |
| azithromycin oral (1 dd. 500 mg) | 0.565 | c, ^1^ |
| erythromycin iv (4 dd. 500mg) | 7.26 | c, ^1^ |
| erythromycin oral (4 dd. 500mg) | 1.35 | c, ^1^ |
| clarithromycin iv (2 dd. 500mg) | 1.15 | c, ^1^ |
| clarithromycin oral (2 dd. 500 mg) | 0.36 | c, ^1^ |
| moxifloxacin iv (1 dd. 400mg) | 63.6 | c, ^1^ |
| moxifloxacin oral (1 dd. 400mg) | 2.715 | c, ^1^ |
| levofloxacin iv (1 dd. 500mg) | 113.78 | c, ^1^ |
| levofloxacin oral (1 dd. 500mg) | 0.86 | c, ^1^ |
| ciprofloxacin iv (2 dd. 400mg) | 20.93 | c, ^1^ |
| ciprofloxacin oral (2 dd. 500mg) | 0.125 | c, ^1^ |
| ofloxacin oral (2 dd. 400mg) | 0.42 | c, ^1^ |
| doxycyclin iv (1 dd. 100 mg (1e dag 200mg)) | 3.72 | c, ^1^ |
| doxycyclin oral (1 dd. 100 mg (1e dag 200mg)) | 0.17 | c, ^1^ |
| vancomycin iv (2 dd. 1g) | 15.195 | c, ^1^ |
| metronidazole iv (4 dd. 500mg) | 3.59 | c, ^1^ |
| metronidazole oral (3 dd. 500mg) | 0.22 | c, ^1^ |
| piperacillin-tazobactam iv (4 dd. 4g/500mg) | 12.55 | c, ^1^ |
| piperacillin iv (4 dd. 4g) | 12.55 | c, ^1^ |
| clindamycin iv (3 dd. 600mg) | 4.89 | c, ^1^ |
| clindamycin oral (4 dd. 300mg) | 0.465 | c, ^1^ |
| flucloxacillin iv (4 dd. 1g) | 4.585 | c, ^1^ |
| flucloxacillin oral (3 dd. 500mg) | 0.14 | c, ^1^ |
| imipenem-cilastatin iv (4 dd. 1g/1g) | 21.535 | c, ^1^ |
| meropenem iv (3 dd. 1g) | 22.42 | c, ^1^ |
| cotrimoxazole iv (2 dd. 960mg) | 2.56 | c, ^1^ |
| cotrimoxazole oral (2 dd. 960mg) | 0.17 | c, ^1^ |
| gentamycin iv (1 dd. 350-400mg) | 9.35 | c, ^1^ |
| tobramycin iv (1 dd. 350-400mg) | 23.997 | c, ^1^ |
| rifampicin iv (1 dd. 600mg) | 6.69 | c, ^1^ |
| rifampicin oral (1 dd. 600mg) | 1.645 | c, ^1^ |
| pheniticillin oral (3 dd. 500mg) | 0.3 | c, ^1^ |
| nitrofurantoin oral (4 dd. 50mg) | 0.105 | c, ^1^ |
| trimethoprim oral (1 dd. 300mg) | 0.2 | c, ^1^ |
| pyrazinamide oral (1 dd. 2g) | 0.66 | c, ^1^ |
| colistin iv (3 dd. 0.5 x10^6^ IE) | 5.06 | c, ^1^ |
| minocycline oral (1 dd. 100mg) | 0.37 | c, ^1^ |
| ethambutol oral (1 dd. 1200mg) | 1.15 | c, ^1^ |
| linezolid iv (2 dd. 600mg) | 59.16 | c, ^1^ |
| ***Other medication costs per day*** |  |  |
| Analgetics: paracetamol (4 dd. 1g) | 0.40 | c, ^2^ |
| Inhaled drugs: salbutamol/ipratropium (4 dd. 0.75/7.5 mg) | 0.90 | c, ^2^ |
| Non-inhaled corticosteroids: prednisolone (4 dd. 10mg) | 0.26 | c, ^2^ |
| vasopressors: norepinephrine (5mg / day) | 4.03 | c, ^2^ |
| ***Hospitalization*** |  |  |
| Hospital admission (general ward) / day | 410.24 | a, ^3^ |
| Intensive care unit |  |  |
| - with artificial ventilation/day | 2699.47 | d |
| - without artificial ventilation/day | 1719.29 | d |
| ***In-hospital medical procedures*** |  | ^4^ |
| Bronchoscopy / broncho-alveolar lavage | 483.97 | d |
| Non-invasive Positive Pressure Ventilation | 291.78 | d |
| Pleural puncture | 146.07 | d |
| Thorax drainage | 969.08 | d |
| Pleurodesis | 1061.09 | d |
| Abscess drainage | 424.21 | d |
| Pacemaker implantation | 8770.00 | d |
| Thoracotomy | 6597.07 | d |
| Lumbar puncture | 316.54 | d |
| Trans-oesophageal echocardiogram | 247.00 | d |
| Chest echography (including cardiac echography) | 72.95 | d |
| Abdominal echography | 107.15 | d |
| Chest CT-scan | 208.60 | d |
| Cranial CT-scan | 192.40 | d |
| CT pulmonary angiogram | 244.42 | d |
| Sinus CT-scan | 192.40 | d |
| Abdominal CT-scan | 208.60 | d |
| Ventilation/perfusion scan | 502.67 | d |
| Video-Assisted Thoracoscopic Surgery | 6761.80 | d |
| Coronary angiography | 296.96 | d |
| Chest X-ray | 96.87 | d |
| ***General practitioner consultations post-discharge*** |  |  |
| Telephone consultation | 14.87 | d |
| Consultation hour visit | 29.73 | d |
| Home visit | 45.66 | d |
| **Medical specialist consultations post-discharge** |  |  |
| Telephone consultation | 38.23 | d |
| Outpatient clinic visit | 76.45 | a |
| Emergency department visit | 160.34 | a |
| Readmission to any hospital / day | 485.28 | a |
| ***Other direct healthcare costs post-discharge*** |  |  |
| Ambulance costs to nursing home |  |  |
| Nursing home / day | 252.73 | a |
| Home care / hour | 37.17 | a |
| ***Direct non-healthcare costs (DNHC)*** |  |  |
| Transport cost/specialist consultation or hospital visit | 4.57 | a, ^5^ |
| Transport cost/GP consultation | 2.06 | a, ^5^ |
| Transport cost/nursing home | 3.16 | a, ^5^ |
| Paid household help (not including home care) | 13.27 | a |
| ***Indirect non-healthcare costs (INHC)*** |  |  |
| *Productivity loss due to absence from:* |  |  |
| Unpaid work / hour | 13.27 | a, ^6^ |
| Paid work (average working person) / hour | 31.88 | a, ^6^ |
| Paid work (Males <20 years) / hour | 10.25 | a |
| Paid work (Females <20 years) / hour | 9.30 | a |
| Paid work (Males 20-24 years) / hour | 18.85 | a |
| Paid work (Females 20-24 years) / hour | 18.24 | a |
| Paid work (Males 25-29 years) / hour | 25.69 | a |
| Paid work (Females 25-29 years) / hour | 25.08 | a |
| Paid work (Males 30-34 years) / hour | 31.48 | a |
| Paid work (Females 30-34 years) / hour | 29.24 | a |
| Paid work (Males 35-39 years) / hour | 36.14 | a |
| Paid work (Females 35-39 years) / hour | 31.06 | a |
| Paid work (Males 40-44 years) / hour | 38.94 | a |
| Paid work (Females 40-44 years) / hour | 30.86 | a |
| Paid work (Males 45-49 years) / hour | 40.69 | a |
| Paid work (Females 45-49 years) / hour | 30.70 | a |
| Paid work (Males 50-54 years) / hour | 41.48 | a |
| Paid work (Females 50-54 years) / hour | 31.06 | a |
| Paid work (Males 55-59 years) / hour | 41.82 | a |
| Paid work (Females 55-59 years) / hour | 31.33 | a |
| Paid work (Males 60-64 years) / hour | 41.55 | a |
| Paid work (Females 60-64 years) / hour | 30.44 | a |
| Paid work (Males >=65 years) / hour | 41.55 | ^7^ |
| Paid work (Females >=65 years) / hour | 30.44 | ^7^ |

1. Prices are given per single standard dosage.
2. For non-antibiotic drugs data on group level were available (e.g. number of days with analgetics). Prices were calculated for the most prescribed drug in this group, based on experience of the authors, and are given per standard defined daily dosage.
3. Medication costs were not included in the daily hospitalization costs of the CAP admission.
4. The following interventions were considered not CAP related and therefore no costs were attributed: liver abscess drainage, abdominal X-ray, PET-CT, dialysis except when patient was on the ICU, and tube feeding.
5. Using average distances as reported in Hakkart et al.,[a] assuming that 50% would use a car and 50% would use public transport.
6. Including productivity loss of a caregiver if reported.
7. No data available for subjects over 65 years of age. If these subjects reported paid work, the productivity loss of subjects 60-64 years of age was used.
8. Hakkaart - van Roijen L, Tan S, Bouwmans C. Methoden en standaard kostprijzen voor economische evaluaties in de gezondheidszorg. 3 ed. Diemen: College van Zorgverzekeringen; 2010.
9. Oosterheert et al. J Clin Microbiol. 2003 Oct;41(10):4708-13.
10. Medication costs derived from <http://www.medicijnkosten.nl/> (Dutch medication costs by “Zorginstituut Nederland” (Health Institute Nederlands)). Prices were looked up in December 2014 and adapted to 2012 euro. Prices are displayed per single standard dose.
11. Mangen et al. Eur Respir J. 2015 Jul 9. pii: ERJ-00325-2015.

**Table S2: Resources used**

|  | **Beta-lactam monotherapy strategy** | **Beta-lactam / macrolide  strategy** | **Fluoroquinolone monotherapy strategy** |
| --- | --- | --- | --- |
| **ADMISSION DAYS** |  |  |  |
| Days in non-ICU ward | 8.1 (7.7 to 8.5) | 9.1 (8.7 to 9.4) | 8.1 (7.8 to 8.5) |
| ICU (%) | 2.7 (1.6 to 4.0) | 1.6 (0.8 to 2.6) | 1.6 (0.9 to 2.4) |
| - Days in ICU (not intubated) | 2.4 (1.3 to 4.1) | 5.1 (1.3 to 10.3) | 2.0 (1.3 to 3.2) |
| - Days in ICU (intubated) | 6.6 (2.4 to 11.1) | 2.6 (1.0 to 4.7) | 5.3 (2.8 to 8.3) |
|  |  |  |  |
| **INTERVENTIONS** |  |  |  |
| Bronchoscopy (%) | 7.3 (5.5 to 9.4) | 7.6 (5.8 to 9.4) | 6.6 (5.0 to 8.5) |
| Broncho-alveolar lavage (%) | 0.0 (0.0 to 0.0) | 0.1 (0.0 to 0.4) | 0.1 (0.0 to 0.3) |
| Non-invasive ventilation (%) | 1.1 (0.4 to 2.0) | 0.6 (0.1 to 1.2) | 1.0 (0.4 to 1.8) |
| Pleural puncture (%) | 4.2 (2.6 to 6.5) | 4.9 (3.3 to 6.9) | 4.1 (2.6 to 5.6) |
| Thorax drainage (%) | 1.2 (0.5 to 2.1) | 2.2 (1.2 to 3.5) | 2.1 (1.2 to 3.2) |
| Pleurodesis (%) | 0.2 (0.0 to 0.5) | 0.0 (0.0 to 0.0) | 0.0 (0.0 to 0.0) |
| Abscess drainage (%) | 0.0 (0.0 to 0.0) | 0.8 (0.3 to 1.5) | 0.3 (0.0 to 0.7) |
| Pacemaker implantation (%) | 0.2 (0.0 to 0.5) | 0.0 (0.0 to 0.0) | 0.0 (0.0 to 0.0) |
| Thoracotomy (%) | 0.0 (0.0 to 0.0) | 0.3 (0.0 to 0.7) | 0.1 (0.0 to 0.3) |
| Lumbar puncture (%) | 0.0 (0.0 to 0.1) | 0.1 (0.0 to 0.4) | 0.2 (0.0 to 0.6) |
| Trans oesophageal echo (%) | 0.2 (0.0 to 0.5) | 0.0 (0.0 to 0.0) | 0.0 (0.0 to 0.0) |
| Echo chest (%) | 0.3 (0.0 to 0.8) | 0.0 (0.0 to 0.0) | 0.0 (0.0 to 0.0) |
| Echo abdomen (%) | 0.2 (0.0 to 0.5) | 0.3 (0.0 to 0.7) | 0.0 (0.0 to 0.0) |
| Echo heart (%) | 0.3 (0.0 to 0.8) | 0.3 (0.0 to 0.7) | 0.6 (0.1 to 1.2) |
| CT thorax (%) | 2.4 (1.3 to 3.8) | 1.4 (0.6 to 2.4) | 0.9 (0.3 to 1.5) |
| CT brain (%) | 0.0 (0.0 to 0.0) | 0.1 (0.0 to 0.4) | 0.0 (0.0 to 0.0) |
| CT angiography (%) | 0.5 (0.0 to 1.2) | 0.1 (0.0 to 0.4) | 0.0 (0.0 to 0.0) |
| CT sinus (%) | 0.0 (0.0 to 0.1) | 0.1 (0.0 to 0.4) | 0.0 (0.0 to 0.0) |
| CT abdomen (%) | 0.0 (0.0 to 0.1) | 0.1 (0.0 to 0.4) | 0.0 (0.0 to 0.0) |
| Ventilation Perfusion scan (%) | 0.2 (0.0 to 0.5) | 0.4 (0.0 to 0.9) | 0.0 (0.0 to 0.0) |
| Video-assisted thoracoscopic surgery(%) | 0.2 (0.0 to 0.5) | 0.1 (0.0 to 0.4) | 0.1 (0.0 to 0.4) |
| Coronary angiography (%) | 0.0 (0.0 to 0.1) | 0.0 (0.0 to 0.0) | 0.1 (0.0 to 0.3) |
| Chest X-ray during admission (%) | 0.0 (0.0 to 0.1) | 0.1 (0.0 to 0.5) | 0.0 (0.0 to 0.0) |
|  |  |  |  |
|  |  |  |  |
| **MEDICATION USE DURING ADMISSION** |  |  |  |
| Analgetics (%) | 44.7 (40.9 to 48.5) | 49.6 (45.8 to 53.4) | 43.4 (39.9 to 47.0) |
| - Days on analgetics | 6.8 (6.1 to 7.9) | 7.1 (6.5 to 7.8) | 6.7 (6.2 to 7.2) |
| Inhalation medication (%) | 48.5 (44.7 to 52.4) | 49.2 (45.7 to 52.5) | 52.0 (49.3 to 55.4) |
| - Days on inhalation medication | 7.3 (6.5 to 8.4) | 7.6 (7.0 to 8.4) | 7.3 (6.9 to 7.8) |
| Systemic corticosteroids (%) | 42.9 (39.3 to 47.1) | 40.5 (37.1 to 43.8) | 45.0 (41.9 to 48.7) |
| - Days on corticosteroids | 7.5 (6.9 to 8.5) | 7.7 (7.1 to 8.2) | 7.7 (7.1 to 8.4) |
| Vasopressors (%) | 0.2 (0.0 to 0.5) | 0.1 (0.0 to 0.4) | 0.6 (0.1 to 1.0) |
| - Days on vasopressors | 4.0 (4.0 to 4.0) | 1.0 (1.0 to 1.0) | 4.2 (1.7 to 6.7) |
|  |  |  |  |
| **ANTIBIOTIC USE DURING ADMISSION** |  |  |  |
| Days with penicillin iv | 0.1 (0.1 to 0.2) | 0.8 (0.7 to 0.9) | 0.3 (0.2 to 0.4) |
| Days with amoxicillin iv | 0.8 (0.7 to 0.9) | 0.3 (0.3 to 0.4) | 0.1 (0.1 to 0.2) |
| Days with amoxicillin oral | 0.9 (0.7 to 1.0) | 0.9 (0.8 to 1.1) | 0.3 (0.2 to 0.4) |
| Days with co-amoxiclav iv | 1.4 (1.2 to 1.5) | 1.1 (0.9 to 1.2) | 0.3 (0.2 to 0.4) |
| Days with co-amoxiclav oral | 1.3 (1.2 to 1.5) | 1.3 (1.2 to 1.5) | 0.4 (0.3 to 0.6) |
| Days with cefuroxime iv | 0.2 (0.1 to 0.3) | 0.6 (0.5 to 0.7) | 0.1 (0.0 to 0.1) |
| Days with ceftriaxone iv | 0.9 (0.7 to 1.1) | 0.9 (0.7 to 1.1) | 0.3 (0.2 to 0.4) |
| Days with cefotaxime iv | 0.0 (0.0 to 0.1) | 0.0 (0.0 to 0.0) | 0.0 (0.0 to 0.0) |
| Days with ceftazidime iv | 0.1 (0.0 to 0.2) | 0.2 (0.1 to 0.3) | 0.1 (0.0 to 0.1) |
| Days with azithromycin oral | 0.2 (0.1 to 0.3) | 0.9 (0.8 to 1.1) | 0.1 (0.0 to 0.1) |
| Days with erythromycin iv | 0.1 (0.1 to 0.2) | 0.9 (0.8 to 1.0) | 0.1 (0.0 to 0.1) |
| Days with erythromycin oral | 0.0 (0.0 to 0.0) | 0.1 (0.1 to 0.2) | 0.0 (0.0 to 0.0) |
| Days with clarithromycin oral | 0.3 (0.2 to 0.5) | 1.4 (1.2 to 1.6) | 0.1 (0.0 to 0.1) |
| Days with moxifloxacin iv | 0.1 (0.0 to 0.1) | 0.0 (0.0 to 0.1) | 1.3 (1.2 to 1.4) |
| Days with moxifloxacin oral | 0.2 (0.1 to 0.3) | 0.1 (0.1 to 0.2) | 1.7 (1.6 to 1.9) |
| Days with levofloxacin iv | 0.0 (0.0 to 0.0) | 0.0 (0.0 to 0.0) | 0.4 (0.4 to 0.5) |
| Days with levofloxacin oral | 0.0 (0.0 to 0.0) | 0.0 (0.0 to 0.0) | 0.9 (0.8 to 1.1) |
| Days with ciprofloxacin iv | 0.2 (0.2 to 0.3) | 0.1 (0.0 to 0.1) | 0.1 (0.0 to 0.1) |
| Days with ciprofloxacin oral | 0.7 (0.6 to 0.9) | 0.5 (0.3 to 0.6) | 0.2 (0.1 to 0.2) |
| Days with doxycyclin iv | 0.0 (0.0 to 0.1) | 0.0 (0.0 to 0.0) | 0.0 (0.0 to 0.0) |
| Days with doxycyclin oral | 0.2 (0.1 to 0.3) | 0.1 (0.1 to 0.2) | 0.1 (0.1 to 0.2) |
| Days with piperacillin-tazobactam iv | 0.1 (0.0 to 0.1) | 0.0 (0.0 to 0.0) | 0.1 (0.0 to 0.2) |
| Days with meropenem iv | 0.0 (0.0 to 0.1) | 0.0 (0.0 to 0.1) | 0.0 (0.0 to 0.1) |
| Days with metronidazole iv | 0.0 (0.0 to 0.0) | 0.0 (0.0 to 0.1) | 0.0 (0.0 to 0.0) |
| Days with clindamycin iv | 0.0 (0.0 to 0.1) | 0.0 (0.0 to 0.1) | 0.0 (0.0 to 0.1) |
| Days with flucloxacillin iv | 0.0 (0.0 to 0.0) | 0.1 (0.0 to 0.1) | 0.1 (0.0 to 0.2) |
| Days with imipenem iv | 0.0 (0.0 to 0.1) | 0.0 (0.0 to 0.0) | 0.0 (0.0 to 0.1) |
| Days with cotrimoxazole oral | 0.1 (0.1 to 0.2) | 0.1 (0.1 to 0.2) | 0.1 (0.0 to 0.1) |
| Days with gentamicin iv | 0.0 (0.0 to 0.1) | 0.0 (0.0 to 0.0) | 0.1 (0.0 to 0.1) |
| Days with tobramycin iv | 0.1 (0.0 to 0.1) | 0.1 (0.0 to 0.1) | 0.0 (0.0 to 0.1) |
| Days with clindamycin oral | 0.0 (0.0 to 0.1) | 0.1 (0.0 to 0.1) | 0.0 (0.0 to 0.1) |
| Days with flucloxacillin oral | 0.0 (0.0 to 0.1) | 0.0 (0.0 to 0.0) | 0.0 (0.0 to 0.0) |
| Days with rifampicin oral | 0.0 (0.0 to 0.0) | 0.0 (0.0 to 0.1) | 0.0 (0.0 to 0.0) |
| Days with cefuroxime oral | 0.0 (0.0 to 0.0) | 0.1 (0.1 to 0.2) | 0.0 (0.0 to 0.0) |
| Days with ceftazidime oral | 0.0 (0.0 to 0.0) | 0.0 (0.0 to 0.0) | 0.0 (0.0 to 0.1) |
| Days with nitrofurantoin oral | 0.0 (0.0 to 0.1) | 0.0 (0.0 to 0.0) | 0.0 (0.0 to 0.0) |
|  |  |  |  |
| **POST DISCHARGE HEALTHCARE USE** |  |  |  |
| GP phone contact (%) | 29.2 (24.5 to 34.0) | 31.8 (26.5 to 37.3) | 31.4 (26.5 to 37.1) |
| - Number of GP phone contacts | 2.7 (2.1 to 3.5) | 2.6 (2.1 to 3.4) | 2.4 (2.0 to 3.0) |
| GP consultation (%) | 24.8 (20.9 to 29.8) | 22.2 (17.9 to 26.8) | 21.5 (18.0 to 25.6) |
| - Number of GP consultations | 1.6 (1.3 to 1.9) | 1.7 (1.4 to 2.1) | 1.7 (1.3 to 2.1) |
| GP home visits (%) | 28.7 (24.2 to 33.1) | 28.7 (23.9 to 34.2) | 30.1 (25.1 to 34.3) |
| - Number of GP home visits | 2.1 (1.7 to 2.5) | 2.0 (1.7 to 2.5) | 2.0 (1.7 to 2.4) |
| Outpatient clinic visit (%) | 28.6 (24.5 to 33.0) | 29.3 (24.2 to 34.2) | 31.4 (27.1 to 35.9) |
| - Number of outpatient clinic visits | 1.7 (1.4 to 2.2) | 1.7 (1.4 to 2.1) | 1.7 (1.4 to 2.1) |
| Readmission (%) | 7.6 (4.5 to 11.7) | 8.4 (5.2 to 13.0) | 7.8 (4.9 to 12.0) |
| - Days readmitted | 9.0 (6.7 to 14.0) | 10.1 (6.9 to 16.2) | 8.9 (6.6 to 12.9) |
| Nursing home admission (%) | 2.9 (1.0 to 5.3) | 3.7 (1.5 to 7.4) | 2.3 (0.8 to 4.6) |
| - Days in nursing home | 21.5 (7.8 to 42.3) | 20.2 (8.5 to 39.3) | 19.7 (7.6 to 40.4) |
| Professional home care (%) | 14.8 (11.0 to 19.1) | 15.5 (11.3 to 20.7) | 15.5 (11.7 to 19.3) |
| - Hours of home care | 10.6 (8.0 to 15.4) | 11.5 (8.3 to 16.1) | 9.8 (7.4 to 13.3) |
|  |  |  |  |
| **POST DISCHARGE ANTIBIOTIC USE** |  |  |  |
| Days with amoxicillin oral pd | 0.7 (0.6 to 0.9) | 0.6 (0.5 to 0.7) | 0.3 (0.2 to 0.3) |
| Days with amoxiclav oral pd | 1.2 (1.0 to 1.7) | 1.3 (0.9 to 2.1) | 0.4 (0.3 to 0.6) |
| Days with cefuroxime oral pd | 0.0 (0.0 to 0.1) | 0.1 (0.1 to 0.2) | 0.0 (0.0 to 0.0) |
| Days with azithromycin oral pd | 0.1 (0.0 to 0.3) | 0.7 (0.2 to 1.8) | 0.0 (0.0 to 0.1) |
| Days with erythromycin oral pd | 0.0 (0.0 to 0.0) | 0.0 (0.0 to 0.1) | 0.0 (0.0 to 0.0) |
| Days with clarithromycin oral pd | 0.2 (0.1 to 0.2) | 0.4 (0.3 to 0.5) | 0.0 (0.0 to 0.1) |
| Days with moxifloxacin oral pd | 0.1 (0.1 to 0.2) | 0.1 (0.1 to 0.2) | 1.6 (1.3 to 2.3) |
| Days with levofloxacin oral pd | 0.0 (0.0 to 0.1) | 0.0 (0.0 to 0.0) | 0.4 (0.3 to 0.5) |
| Days with ciprofloxacin oral pd | 0.3 (0.2 to 0.5) | 0.1 (0.1 to 0.3) | 0.6 (0.1 to 1.4) |
| Days with doxycyclin oral pd | 0.1 (0.0 to 0.2) | 0.1 (0.0 to 0.2) | 0.1 (0.0 to 0.1) |
| Days with cotrimoxazole oral pd | 0.1 (0.0 to 0.2) | 0.1 (0.0 to 0.3) | 0.0 (0.0 to 0.1) |
| Days with clindamycin oral pd | 0.1 (0.0 to 0.2) | 0.2 (0.0 to 0.3) | 0.2 (0.0 to 0.3) |
| Days with flucloxacillin oral pd | 0.0 (0.0 to 0.0) | 0.0 (0.0 to 0.0) | 0.0 (0.0 to 0.1) |
| Days with pheniticillin oral pd | 0.0 (0.0 to 0.0) | 0.0 (0.0 to 0.1) | 0.0 (0.0 to 0.0) |
| Days with linezolid iv pd | 0.0 (0.0 to 0.0) | 0.0 (0.0 to 0.0) | 0.0 (0.0 to 0.1) |
|  |  |  |  |
| **PATIENT COSTS** |  |  |  |
| Patient declared costs (Euro) | 129.2 (96.3 to 175.2) | 128.6 (100.3 to 168.2) | 119.7 (92.1 to 161.0) |
|  |  |  |  |
| **PRODUCTIVITY LOSS** |  |  |  |
| Paid work (%) | 21.6 (18.1 to 25.7) | 22.8 (19.0 to 26.4) | 22.7 (19.2 to 26.2) |
| - Weekly hours of paid work | 31.7 (29.1 to 34.9) | 31.6 (29.0 to 34.9) | 31.1 (28.7 to 34.3) |
| Volunteer work (%) | 12.4 (9.4 to 16.1) | 14.9 (11.3 to 19.6) | 13.6 (10.6 to 17.8) |
| - Weekly hours of volunteer work | 10.0 (7.6 to 12.9) | 9.6 (7.4 to 12.9) | 10.6 (8.1 to 14.1) |
| Paid work loss of patient (hours) | 22.2 (18.1 to 27.7) | 23.3 (19.1 to 29.6) | 22.8 (18.3 to 27.8) |
| Volunteer work loss of patient (hours) | 5.2 (3.1 to 8.0) | 6.2 (3.9 to 9.2) | 6.0 (3.6 to 9.4) |
| Paid work loss of caregiver (hours) | 4.0 (3.0 to 5.4) | 4.2 (2.9 to 5.9) | 4.1 (3.0 to 5.7) |
| Volunteer work loss of caregiver (hours) | 1.1 (0.4 to 2.0) | 1.0 (0.4 to 1.8) | 1.1 (0.4 to 1.8) |
| 90-day mortality working population (%) | 6.1 (2.1 to 13.5) | 5.6 (2.1 to 12.1) | 4.9 (1.7 to 10.7) |
| 30-day mortality working population (%) | 3.8 (0.6 to 10.9) | 2.8 (0.3 to 9.1) | 3.0 (0.5 to 8.3) |

Data represent the point estimate and 95% confidence interval of the mean or proportion, as indicated. These were derived from the 50^th^, 2.5^th^ and 97.5^th^ percentiles of the 2,000 bootstrapped datasets following 5 imputations in each dataset.

**Table S3: Cost and effect estimates**

|  | **Beta-lactam  monotherapy  strategy** | **Beta-lactam/macrolide  combination  strategy** | **Fluoroquinolone  monotherapy  strategy** |
| --- | --- | --- | --- |
| **90-day time horizon** |  |  |  |
| All-cause mortality | 9.1% (7.0% to 11.6%) | 11.1% (8.9% to 13.5%) | 8.8% (6.9% to 10.5%) |
| *Costs (€/patient)* |  |  |  |
| Reduced third payer | 4,294 (3,782 to 4,952) | 4,392 (4,062 to 4,760) | 4,002 (3,725 to 4,341) |
| Third payer | 4,959 (4,372 to 5,682) | 5,170 (4,759 to 5,627) | 4,641 (4,270 to 5,033) |
| Societal (friction) | 5,972 (5,322 to 6,772) | 6,184 (5,697 to 6,747) | 5,668 (5,249 to 6,128) |
| Societal (human capital) | 7,554 (5,842 to 11,134) | 6,700 (5,906 to 8,468) | 6,891 (5,621 to 9,463) |
| *CMA (Difference(∆) in costs/treated patient) Ɨ* |  |  |  |
| Reduced third payer | [reference] | 106 (-697 to 754) | -278 (-991 to 396) |
| Third payer | [reference] | 210 (-722 to 911) | -279 (-1,094 to 464) |
| Societal (friction) | [reference] | 216 (-725 to 1,032) | -277 (-1,246 to 539) |
| Societal (human capital) | [reference] | -964 (-4,967 to 1,614) | -865 (-4,806 to 2,247) |
|  |  |  |  |
| **30-day time horizon** |  |  |  |
| All-cause mortality | 5.5% (4.0% to 7.3%) | 5.8% (4.3% to 7.5%) | 4.9% (3.5% to 6.3%) |
| *Costs (€/patient)* |  |  |  |
| Reduced third payer | 4,161 (3,727 to 4,668) | 4,316 (4,035 to 4,632) | 3,925 (3,683 to 4,231) |
| Third payer | 4,649 (4,169 to 5,164) | 4,835 (4,508 to 5,179) | 4,395 (4,105 to 4,700) |
| Societal (friction) | 5,586 (5,023 to 6,193) | 5,744 (5,395 to 6,136) | 5,386 (5,039 to 5,779) |
| Societal (human capital) | 6,487 (5,247 to 9,726) | 5,748 (5,395 to 6,136) | 6,077 (5,213 to 8,571) |
| *CMA (Difference(∆) in costs/treated patient)* Ɨ |  |  |  |
| Reduced third payer | [reference] | 159 (-459 to 679) | -200 (-798 to 352) |
| Third payer | [reference] | 184 (-447 to 724) | -197 (-820 to 365) |
| Societal (friction) | [reference] | 175 (-598 to 799) | -133 (-926 to 548) |
| Societal (human capital) | [reference] | -842 (-4,522 to 499) | -362 (-4,084 to 2,122) |
| Ɨ As the primary effect (i.e. case-fatality) was not significantly different,[9] a cost-minimization analysis (CMA) was applied. CMA is expressed as the difference in costs using mixed-effects linear regression analysis with beta-lactam monotherapy as the reference strategy. Cost-effectiveness analysis (CEA) was conducted as well, and as to be expected (i.e. no significant difference in primary results) ranged from being dominated to cost-saving. CEA results were therefore presented as cost-effectiveness plots in the Figures S1, S2 and S3. | | | |

**Figure S1: Cost-effectiveness plots from a third payer perspective**

| - 1. Beta-lactam/macrolide strategy vs. beta-lactam strategy – 90-day time horizon   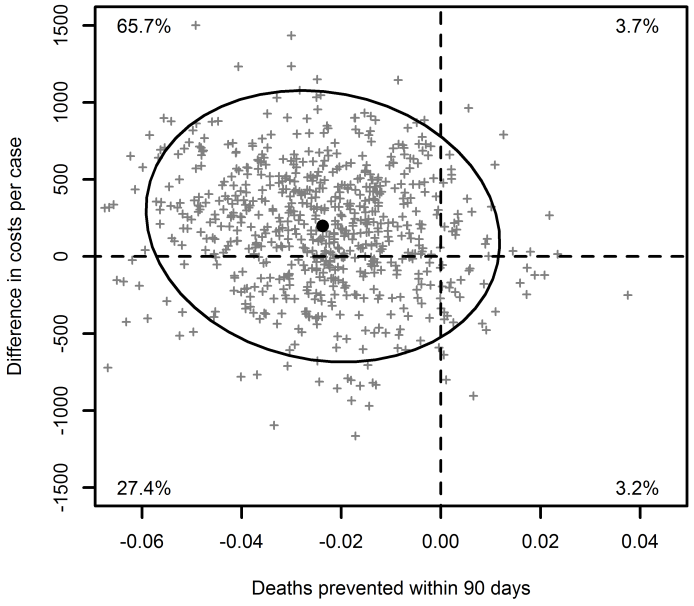 | - 1. Beta-lactam/macrolide strategy vs. beta-lactam strategy – 30-day time horizon   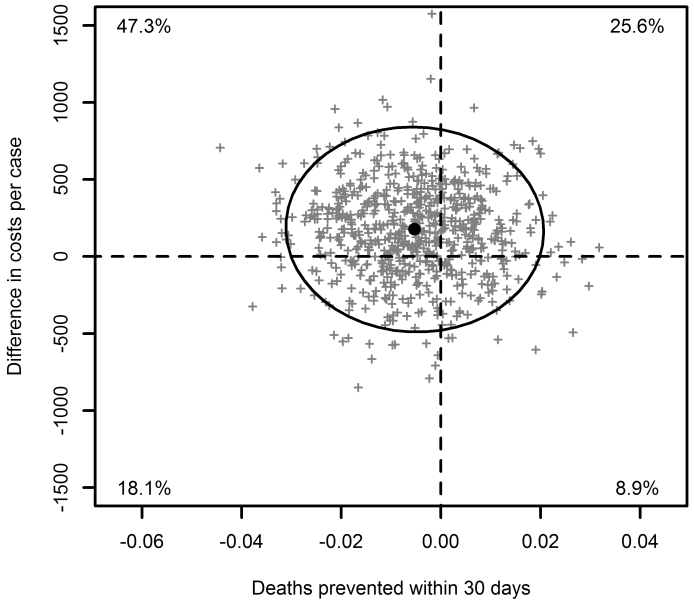 |
| --- | --- |
| - 1. Fluoroquinolone strategy vs. beta-lactam strategy – 90-day time horizon   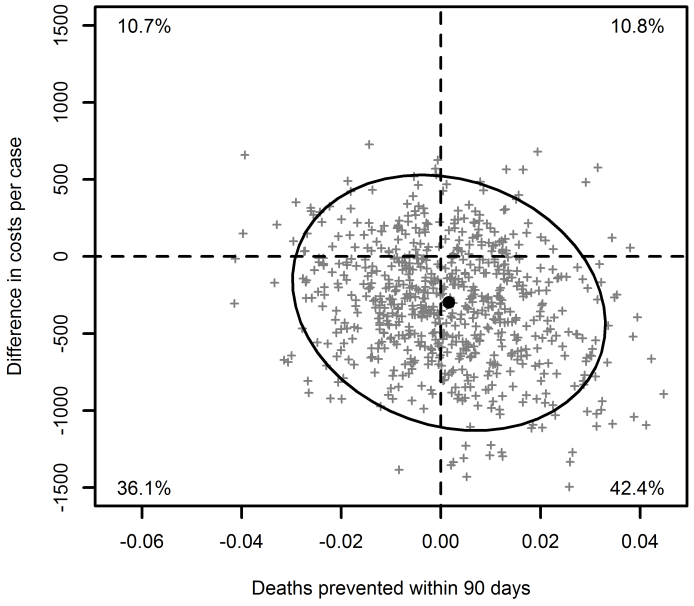 | - 1. Fluoroquinolone strategy vs. beta-lactam strategy – 30-day time horizon   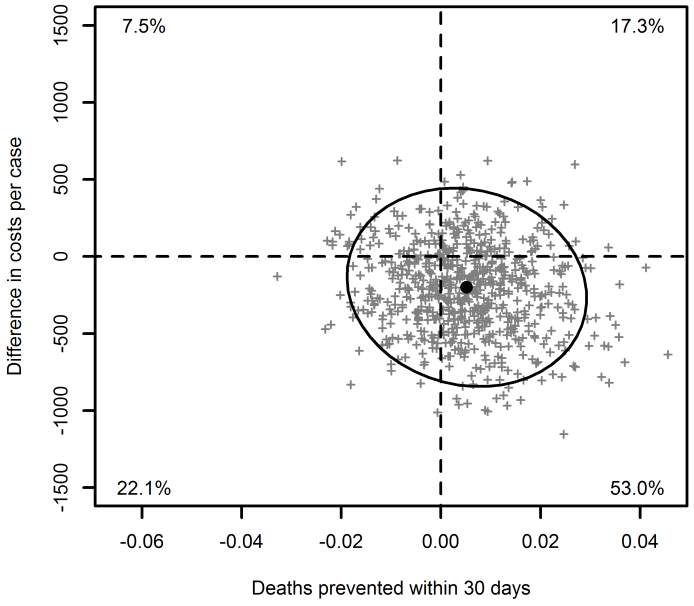 |

Grey points represent incremental costs and incremental effects of 2,000 bootstrapping samples for the beta-lactam/macrolide combination strategy compared to the beta-lactam monotherapy strategy within 90 (A) and 30 (B) days of admission, and for the fluoroquinolone monotherapy strategy compared to the beta-lactam monotherapy strategy within 90 (C) and 30 (D) days of admission. The black points and curves represent the point estimates and the 95% confidence ellipses. Proportions in each quadrant indicate the proportion of bootstrap samples in that quadrant. Point estimates in the north-west quadrant are in favour of the beta-lactam monotherapy strategy; point estimates in the south-east quadrant are in favour of the other strategy. Exact point estimates and 95% confidence intervals for incremental costs and incremental effects are given in Supplementary Appendix Table S3.

**Figure S2: Cost-effectiveness plots from a societal perspective, friction approach**

| 1. Beta-lactam/macrolide strategy vs. beta-lactam strategy – 90-day time horizon   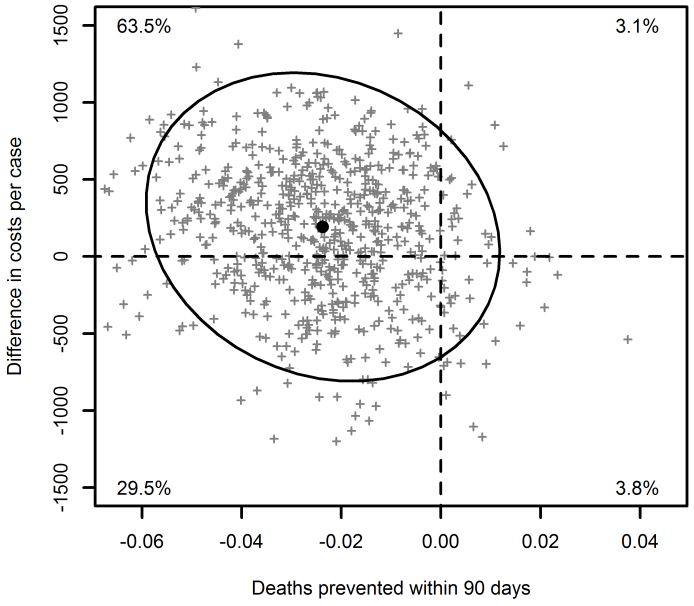 | 1. Beta-lactam/macrolide strategy vs. beta-lactam strategy – 30-day time horizon   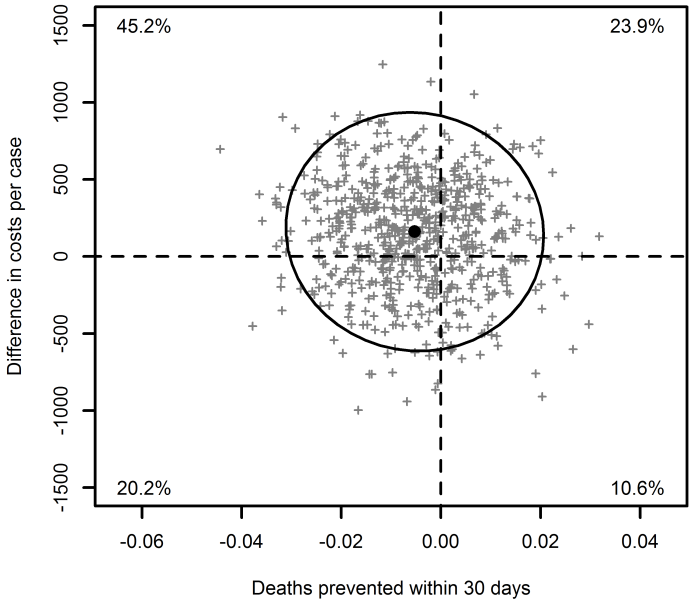 |
| --- | --- |
| 1. Fluoroquinolone strategy vs. beta-lactam strategy – 90-day time horizon   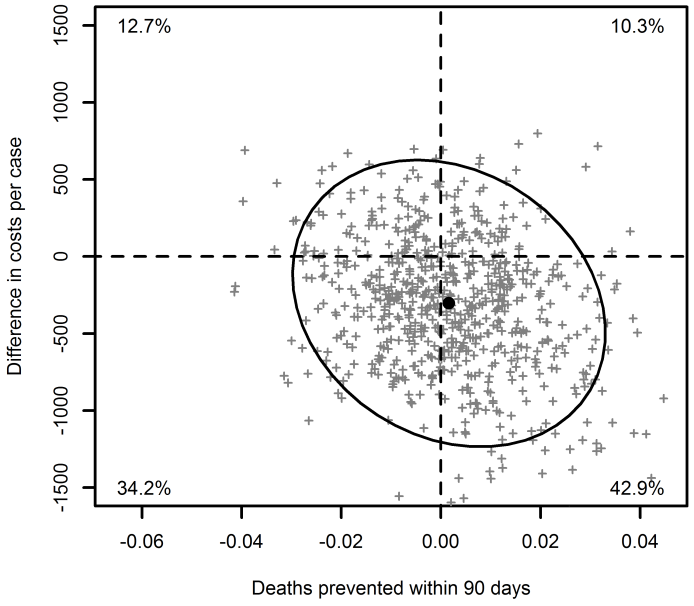 | 1. Fluoroquinolone strategy vs. beta-lactam strategy – 30-day time horizon   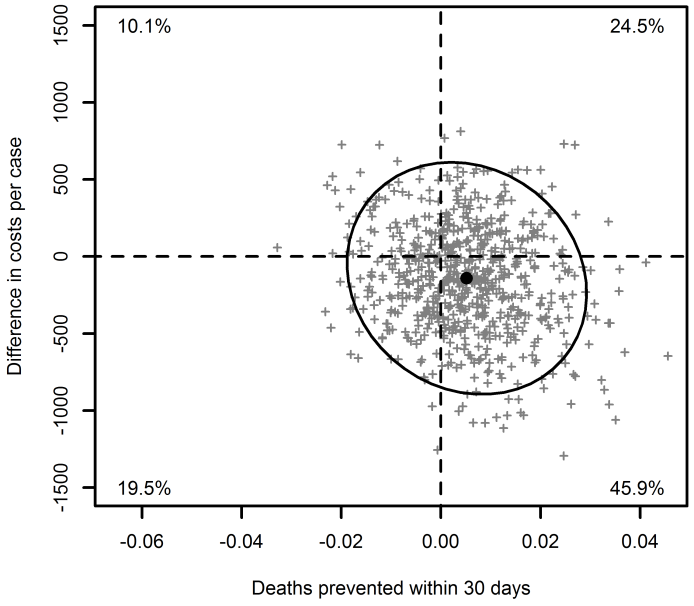 |

Grey points represent incremental costs and incremental effects of 2,000 bootstrapping samples for the beta-lactam/macrolide combination strategy compared to the beta-lactam monotherapy strategy within 90 (A) and 30 (B) days of admission, and for the fluoroquinolone monotherapy strategy compared to the beta-lactam monotherapy strategy within 90 (C) and 30 (D) days of admission. The black points and curves represent the point estimates and the 95% confidence ellipses. Proportions in each quadrant indicate the proportion of bootstrap samples in that quadrant. Point estimates in the north-west quadrant are in favour of the beta-lactam monotherapy strategy; point estimates in the south-east quadrant are in favour of the other strategy. Exact point estimates and 95% confidence intervals for incremental costs and incremental effects are given in Supplementary Appendix Table S3.

**Figure S3: Cost-effectiveness plots from a societal perspective, human capital approach**

| - 1. Beta-lactam/macrolide strategy vs. beta-lactam strategy – 90-day time horizon   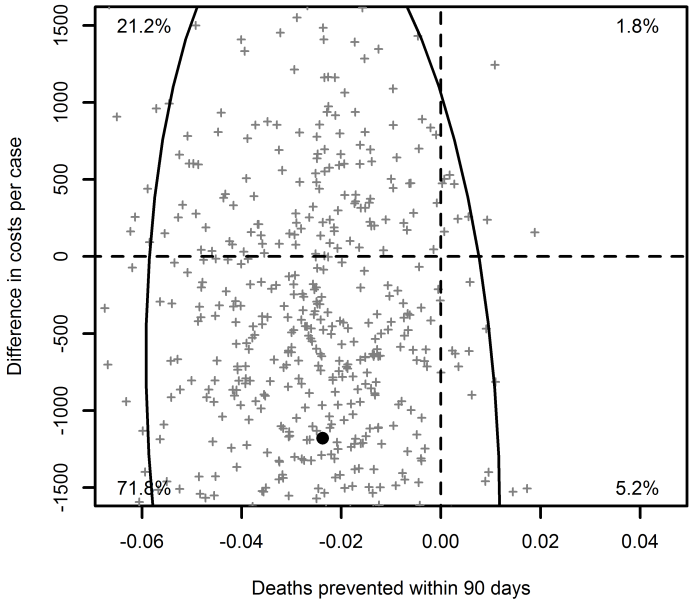 | - 1. Beta-lactam/macrolide strategy vs. beta-lactam strategy – 30-day time horizon   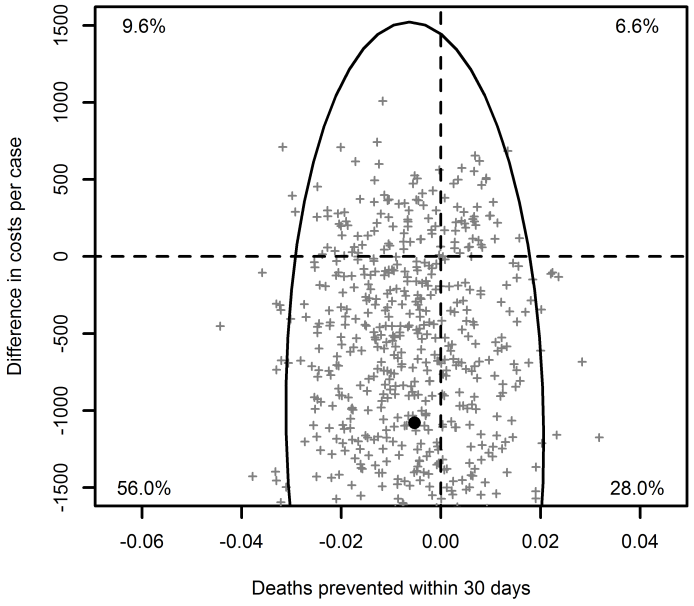 |
| --- | --- |
| - 1. Fluoroquinolone strategy vs. beta-lactam strategy – 90-day time horizon   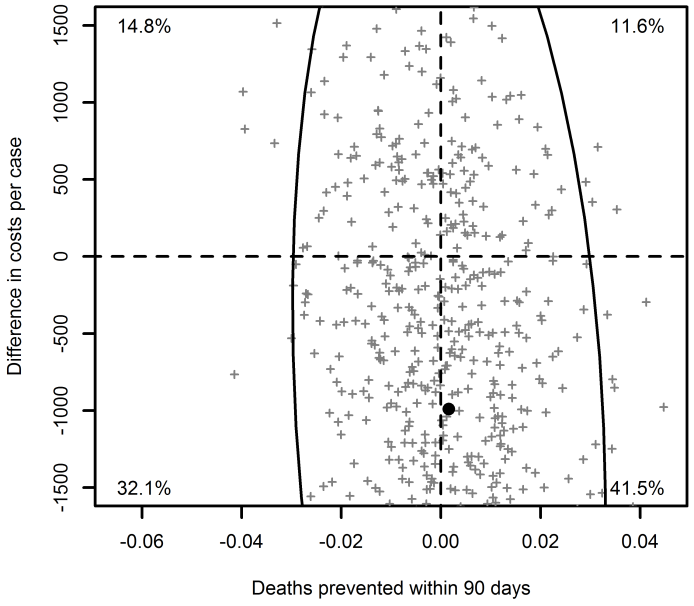 | - 1. Fluoroquinolone strategy vs. beta-lactam strategy – 30-day time horizon   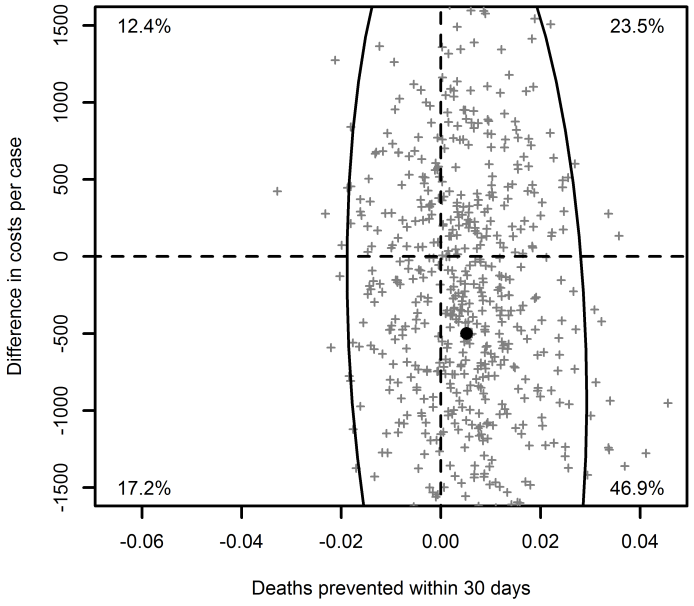 |

Grey points represent incremental costs and incremental effects of 2,000 bootstrapping samples for the beta-lactam/macrolide combination strategy compared to the beta-lactam monotherapy strategy within 90 (A) and 30 (B) days of admission, and for the fluoroquinolone monotherapy strategy compared to the beta-lactam monotherapy strategy within 90 (C) and 30 (D) days of admission. The black points and curves represent the point estimates and the 95% confidence ellipses. Proportions in each quadrant indicate the proportion of bootstrap samples in that quadrant. Point estimates in the north-west quadrant are in favour of the beta-lactam monotherapy strategy; point estimates in the south-east quadrant are in favour of the other strategy. Exact point estimates and 95% confidence intervals for incremental costs and incremental effects are given in Supplementary Appendix Table S3.
